# The Effects of Various Weather Conditions as a Potential Ischemic Stroke Trigger in Dogs

**DOI:** 10.3390/vetsci4040056

**Published:** 2017-11-16

**Authors:** Kristy L. Meadows, Gena M. Silver

**Affiliations:** 1 Cummings School of Veterinary Medicine, Tufts University, 200 Westboro Rd., Grafton, MA 01536, USA; 2 Massachusetts Veterinary Referral Hospital, 20 Cabot Rd., Woburn, MA 01801, USA; gsilver@ethosvet.com

**Keywords:** canine, ischemic stroke, stroke trigger, weather

## Abstract

Stroke is the fifth leading cause of death in the United States, and is the leading cause of serious, long-term disability worldwide. There are at least 795,000 new or recurrent strokes each year, and approximately 85% of all stroke occurrences are ischemic. Unfortunately, companion animals are also at risk for ischemic stroke. Although the exact incidence of ischemic stroke in companion animals is unknown, some studies, and the veterinary information network (VIN), report that approximately 3% of neurological case referrals are due to a stroke. There is a long list of predisposing factors associated with the risk of ischemic stroke in both humans and canines; however, these factors do not explain why a stroke happens at a particular time on a particular day. Our understanding of these potential stroke “triggers” is limited, and the effect of transient environmental exposures may be one such “trigger”. The present study investigated the extent to which the natural occurrence of canine ischemic stroke was related to the weather conditions in the time-period immediately preceding the onset of stroke. The results of the present study demonstrated that the change in weather conditions could be a potential stroke trigger, with the strokes evaluated occurring after periods of rapid, large fluctuations in weather conditions. There are currently no epidemiological data on the seasonal variability of ischemic stroke in dogs, and determining whether canine stroke parallels human stroke would further validate the use of companion dogs as an appropriate naturally occurring model.

## 1. Introduction

Stroke is the fifth leading cause of death in the United States behind heart disease, cancer, chronic lower respiratory disease, and general accidents, and is the leading cause of chronic, functional incapacity and serious, long-term disability worldwide [1,2,3,4,5,6]. There are at least 795,000 new or recurrent strokes each year, and approximately 85% of all stroke occurrences are ischemic [1,2,6,7]. Unfortunately, companion animals are also at risk for ischemic stroke. Although the exact incidence of ischemic stroke in companion animals is unknown, some studies, and the veterinary information network (VIN), report that approximately 3% of neurological case referrals, both canine and feline, are presumed to be due to a cerebrovascular accident [8,9,10].

There is a long list of predisposing factors associated with the risk of ischemic stroke in both humans and canines, including but not limited to increasing age, hypertension, type II diabetes, obesity, high cholesterol, hyperlipidemia, hyperadrenocorticism, hypothyroidism, heart disease, and chronic kidney disease [1,2,7,9,11,12,13]. The effects of these predisposing factors are fairly stable, contributing to an overall higher risk of stroke; however, these factors do not explain why a stroke happens to a particular individual at a particular time on a particular day. Our understanding of potential stroke “triggers” is limited, and their study is becoming increasingly important. In order to fully understand the complex, multifactorial event that is ischemic stroke, one must consider the well-known traditional risk factors, but also the effects of transient environmental exposures. One such exposure is the effect of weather, including ambient temperatures, atmospheric pressure, and relative humidity, among others.

The mechanisms underlying the relationship between weather parameters and the risk of ischemic stroke are not well understood. However, there are certain physiologic parameters that may be associated with increased stroke risk that display variations based on weather patterns. For example, one of the most common risk factors for ischemic stroke is hypertension [1,2,3,4], and it is commonly reported that vasoconstriction and blood pressure display a winter peak, and that blood pressure increases with decreasing temperatures [5,6,7,8,9,10]. In addition, it has been shown that cold can activate the sympathetic nervous system, thereby inducing the production of norepinephrine and epinephrine, which both increase blood pressure [8,9,11,12]. Furthermore, it has been shown that cold weather can increase circulating cholesterol, triglycerides, fibrinogen, Factor VII, red blood cells, and platelets, all of which can contribute to a hypercoagulable state, which is another risk factor for ischemic stroke [5,6,7,8,10,13]. 

The results of previous studies examining the weather as a potential stroke trigger in humans are varied, with the majority of studies reporting that decreases in temperature may be an important trigger, and that stroke incidence and mortality tend to display a winter peak. For example, the Framingham Heart Study found that there was a winter peak in ischemic strokes, with January being the most frequent month of onset [14]. Investigators in Boston, Massachusetts found that the risk of ischemic stroke increased if there was a 5 °C decrease in temperature over the two days prior to stroke onset, even after adjusting for risk factors like age, sex, smoking, type II diabetes, hypertension, and atrial fibrillation [15]. Some studies show that it is not the direction of the change in temperature that is important, but the rapid change itself that may be the trigger. For example, in a large, nationally representative sample of 171,695 ischemic stroke patients in the United States, it was demonstrated that lower daily average temperatures and large daily variations of greater than 5 °C were associated with an increased risk of stroke associated hospitalizations, particularly in the North East in the spring and fall [16]. Lastly, a recent systematic review and meta-analysis, encompassing 20 articles, found a positive relationship between a 1 °C change in temperature and the occurrence of major adverse cerebrovascular events [17]. In fact, it was demonstrated that the occurrence of a major adverse cerebrovascular event increased by 1.1% when the temperature increased, and increased by 1.2% when the temperature decreased [17].

The study of the weather as a potential stroke trigger in humans is controversial, as the weather cannot be controlled or studied systematically, and there have been studies that have concluded that there is virtually no association between stroke incidence and any weather parameter. For example, a large analysis of 196,439 stroke-associated admissions across 155 hospitals in the United States found no seasonal change in admissions and no association between any variable and any stroke subtype [18]. The disparate results found across the literature highlights our relatively limited understanding regarding weather parameters as stroke triggers, and emphasizes the need for further study.

Studying naturally occurring ischemic stroke in dogs has several advantages over traditional rodent models. Spontaneously occurring ischemic stroke in dogs is similar to human ischemic stroke in both clinical symptoms and infarct topography [19,20,21,22,23]. In addition, companion dogs live a full lifespan in which they may also suffer several comorbidities, including diabetes, heart disease and/or, various inflammatory conditions [19,20,21,22,23]. Companion dogs share the same environment with humans, have access to similar high quality medical care, and have a similarly sized, gyrencephalic brain, which receives its blood supply through a well-defined arterial Circle of Willis resembling that of humans [19,20,21,22,23,24,25]. Furthermore, the speed at which dogs age, combined with the lesser regulations imposed upon companion animal studies, can accelerate the rate at which small animal clinical trials can provide information regarding novel treatment options, providing an advantage over studying humans themselves [14,15]. Hence, companion dogs represent a ready clinical population that can be used to investigate potential stroke triggers, especially transient environmental exposures like the weather.

The goal of the current study was to determine the extent to which the natural occurrence of canine ischemic stroke is related to prevailing weather conditions (temperature, atmospheric pressure, and humidity) in the time-period immediately preceding each event. Specifically, we hypothesized that the incidence of canine ischemic stroke will be increased with decreases in temperature, and rapid changes in atmospheric pressure and humidity. Moreover, there are currently no epidemiological data on the seasonal variability of ischemic stroke in dogs, and determining if canine stroke parallels human stroke could validate the use of companion dogs as an appropriate naturally occurring model.

## 2. Materials and Methods

### 2.1. Canine Data Collection

Medical records from dogs presenting to IVG Massachusetts Veterinary Referral Hospital (Woburn, MA, USA) were collected. Records from dogs where advanced imaging with MRI displayed an intraparenchymal hyperintensity with relatively sharp borders, consistent with ischemic stroke were used. Pre-contrast images of the brain using the following sequences were evaluated: sagittal, transverse, and dorsal T2w TSE, T2w FLAIR, and T1w SE. Following contrast administration, T1w SE images in transverse, sagittal, and dorsal planes were evaluated. All images were evaluated by a single board certified veterinary neurologist who was not blinded to the clinical condition of the patient. In addition, the medical records from all dogs included in the current study were evaluated for clinical course post-stroke, and all demonstrated either a static progression of clinical signs or improvement. Table 1 displays the canine patient demographics.

### 2.2. Meteorological Exposures

Data on temperature, atmospheric pressure, and relative humidity were obtained from the National Centers for Environmental Information National Oceanic and Atmospheric Administration (NOAA), which compiles data for this region from a single weather station at Boston Logan International Airport [26].

### 2.3. Statistical Analysis

The current study had a case–crossover design, which helps to lower between-subject variability, as the patient serves as their own control [15,27,28]. Each patient has a case window, or period of time where the patient was a case. The case window in the current study was defined as the day on which the stroke occurred, as well as the seven days prior to stroke occurrence (7-day hazard periods (H) before and including the canine stroke events). In addition, each patient has a control window, or a period of time where the patient was not associated with being a case. The control window in the current study was defined as the eighth day prior to stroke occurrence (1-day control period (C) before the canine stroke events). Risk exposure to a transient event during the case window is compared to risk exposure during the control window. An analysis was performed to determine the extent to which 15 occurrences of canine stroke, recorded on the dates listed in Table 2, were related to the prevailing weather conditions (temperature, atmospheric pressure, and humidity) in the time-period immediately preceding each stroke. All plots were constructed and data analyzed with Minitab 17 (Minitab Inc., State College, PA, USA) unless otherwise specified. 

Chi-square analysis was performed from the frequency distribution of the observed canine ischemic strokes with GraphPad Prism (GraphPad Software Inc., La Jolla, CA, USA).

Furthermore, time series plots of mean temperature (°C), mean atmospheric pressure (mmHg), and mean humidity (%) prior to the 15 canine strokes were constructed where Day 8 is the day on which the stroke occurred, and Days 1 to 7 are the 7 days preceding the stroke. Days 2 to 8 were classified as the hazard period (H) and Day 1 as the control period (C). 

The sample size (*n* = 15 dogs) was too small to provide sufficient power to conduct multivariate statistics to compare the control and the hazard periods, and the publication of research containing the erroneous results of underpowered statistical analysis is generally considered to be unethical conduct [29,30,31]. Therefore, a simpler approach was applied. Error bar charts displaying the mean temperature (°C), mean atmospheric pressure (mmHg), and mean humidity (%) in the one-day control periods (C) and the mean temperature (°C), mean atmospheric pressure (mmHg), and mean humidity (%) ± the 95% confidence intervals (CI) in the seven-day hazard periods (H) before the 15 canine strokes were constructed. The method used is the same used by other researchers to determine the effects of weather on the incidence of stroke [15,27,28,32,33]. Using a recognized method that has been described in the literature can help to make the results comparable with previous studies. If the mean temperatures, mean atmospheric pressures, or mean humidities in the control period were not strongly captured within the 95% confidence intervals of the hazard periods, then a significant difference between the mean values at the *p* < 0.05 level of significance was likely [34,35]. Recent publications have recommended the interpretation of means with 95% confidence intervals, and effect sizes, as a more meaningful explanation of results when compared to *p*-values [36,37]. In addition, the apparent temperature calculation, which combines temperature, wind speed, and relative humidity into one measure, was compared using the same method described above [15,38,39]. The apparent temperature (AT) equation is AT = Ta + 0.33 × e − 0.70 × ws − 4.0, where Ta is air temperature (°C), e is water vapor pressure, and ws is wind speed (m/s) [15,38,39]. Water vapor pressure (e) was calculated using the equation e = rh/100 × 6.105 × exp(17.27 × Ta/(237.7 + Ta)), where rh is relative humidity (%) [15,38,39]. 

## 3. Results

As shown in Table 2, the weather conditions in the 8 days preceding each stroke occurrence were extremely variable. The mean daily temperatures ranged from −9.31 to 22.08 °C, the mean daily atmospheric pressures (at sea level) ranged from 757.49 to 767.14 mmHg, and the mean daily humidity ranged from 62.50 to 80.88%.

**Figure 1:**
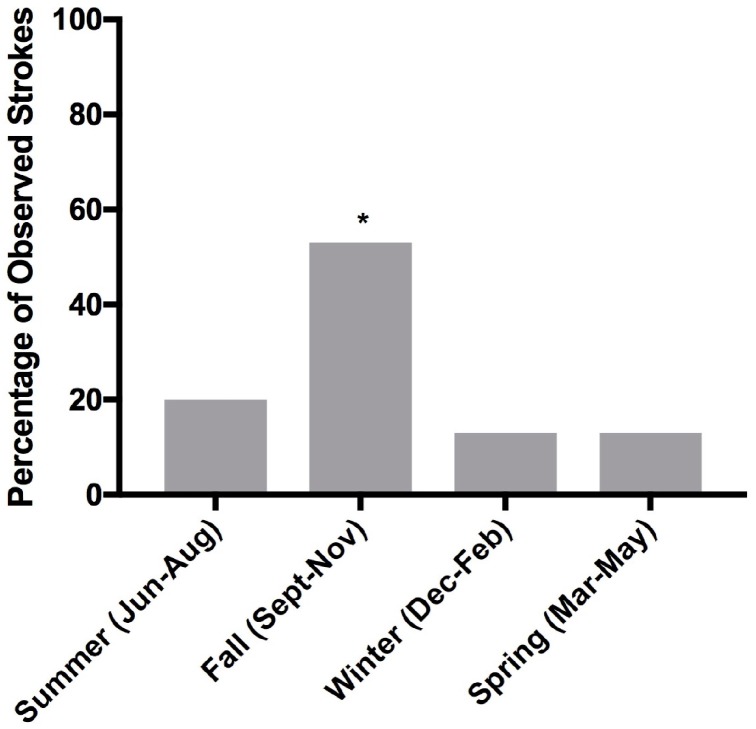
Histogram of the percentage of observed canine ischemic strokes (*n* = 15) between 2011 and 2015 by season (chi-square, df; 18.18,3 * *p* < 0.01).

Figure 1 shows that the occurrence of 15 canine ischemic strokes between 2011 and 2015 display an obvious peak in the fall season (September–November) as demonstrated by a chi-square test (chi-square, df: 18.18,3; *p* < 0.01).

**Figure 2:**
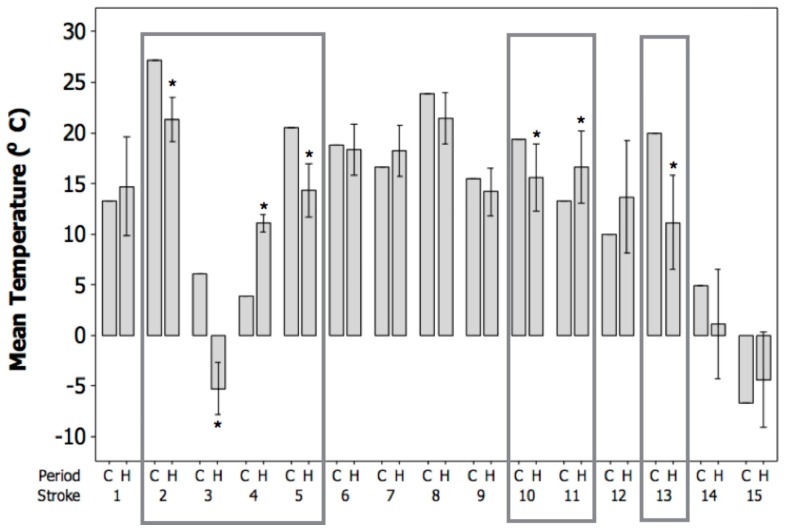
Mean temperature (°C) in the control periods (C) and the mean temperature ±95% CI in the 7-day hazard periods (H) before the canine strokes (*n* = 15). Significant effects (* *p* < 0.05) are displayed within boxes.

Figure 2 displays the mean temperature (°C) in the control period (C) and the mean temperature ± 95% CI in the 7-day hazard periods (H) before the canine stroke events. Before two of the strokes (numbered 4 and 11), the mean temperatures during the control periods were significantly lower than during the hazard periods, reflecting that the strokes occurred after a rapid increase in temperature. Before five of the strokes (numbered 2, 3, 5, 10, and 13), the mean temperatures in the control periods were significantly higher than during the hazard periods reflecting that the strokes occurred after a rapid decrease in temperature. Before eight of the strokes (numbered 1, 6, 7, 8, 9, 12, 14, and 15), there was no significant difference between the mean temperature in the control period and the hazard period. Rapid, large changes in mean temperature in either direction occurred in 7 out of the 15 observed canine strokes (47%), with an average change of 4.5 °C.

**Figure 3:**
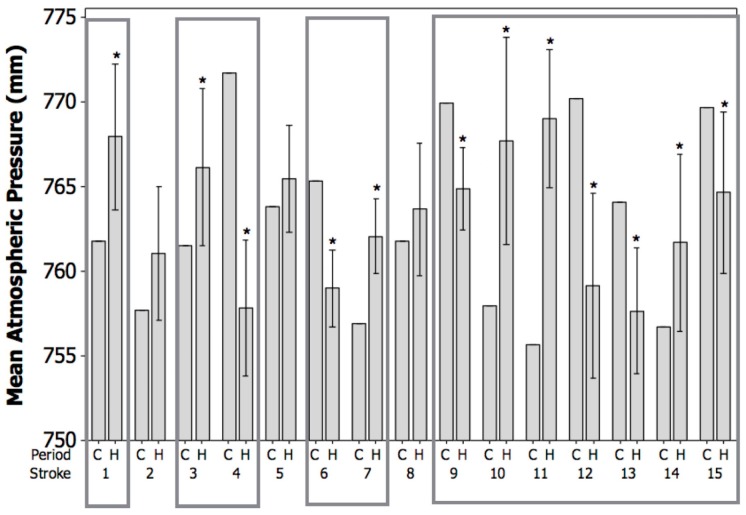
Mean atmospheric pressure (mmHg) in the control period (C) and the mean atmospheric pressure ±95% CI in the 7-day hazard periods (H) before the canine strokes (*n* = 15). Significant effects (* *p* < 0.05) are displayed within boxes.

Figure 3 displays the mean atmospheric pressure (mmHg) in the control period (C) and the mean atmospheric pressure ±95% CI in the 7-day hazard periods (H) before the canine stroke events. Before six of the strokes (numbered 1, 3, 7, 10, 11, and 14), there was a significant increase in the mean atmospheric pressure between the control period and the hazard period, indicating that the strokes occurred after a rapid increase in pressure. Before six of the strokes (numbered 4, 6, 9, 12, 13, and 15), there was a significant decrease in the mean atmospheric pressure between the control period and the hazard period, indicating that the strokes occurred after a rapid decrease in pressure. Before three of the strokes (numbered 2, 5, and 8), there was no significant difference between the mean atmospheric pressure in the control period and the hazard period. Rapid, large changes in mean atmospheric pressure in either direction occurred in 12 out of the 15 observed canine strokes (80%), with an average change of 6.8 mmHg.

**Figure 4:**
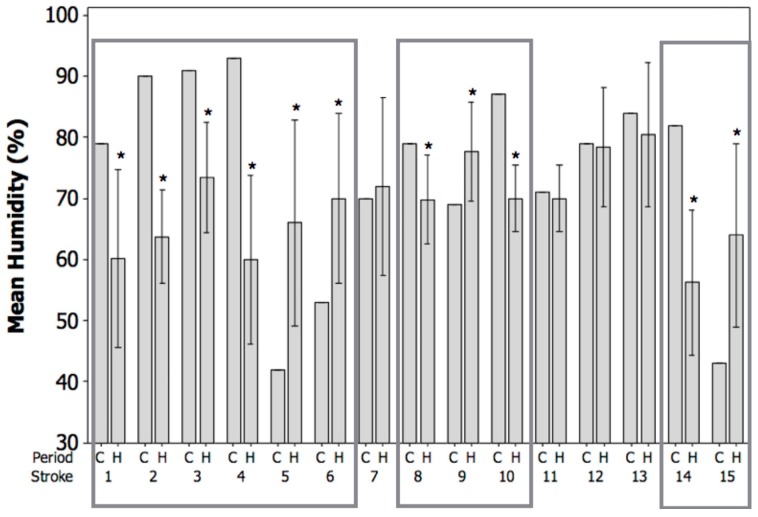
Mean humidity (%) in the control period (C) and the mean humidity ±95% CI in the 7-day hazard periods (H) before the canine strokes (*n* = 15). Significant effects (* *p* < 0.05) are displayed within boxes.

Figure 4 displays the mean humidity (%) in the control period (C) and the mean humidity ±95% CI in the 7-day hazard periods (H) before the canine stroke events. Before four of the strokes (numbered 5, 6, 9, and 15), there was a significant increase in the mean humidity between the control period and the hazard period, indicating that the strokes occurred after a rapid increase in humidity. Before seven of the strokes (numbered 1, 2, 3, 4, 8, 10, and 14), there was a significant decrease in the mean humidity between the control period and the hazard period, indicating that the strokes occurred after a rapid decrease in humidity. Before four of the strokes (numbered 7, 11, 12, and 13), there was no significant difference between the mean humidity in the control period and the hazard period. Rapid, large changes in mean humidity in either direction occurred in 11 out of the 15 observed canine strokes (73%), with an average change of 15%. 

**Figure 5:**
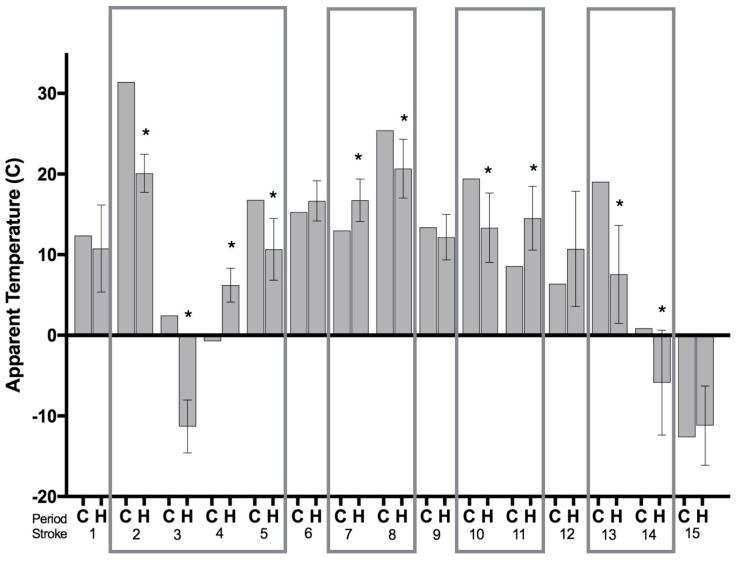
Mean apparent temperature (°C) in the control period (C) and the mean apparent temperature ±95% CI in the 7-day hazard periods (H) before the canine strokes (*n* = 15). Significant effects (* *p* < 0.05) are displayed within boxes.

Figure 5 displays the mean apparent temperature (°C) in the control periods (C) and the mean apparent temperature ±95% CI in the 7-day hazard periods (H) before the canine stroke events. Before three of the strokes (numbered 4, 7, and 11), the mean apparent temperatures during the control periods were significantly lower than during the hazard periods, reflecting that the strokes occurred after a rapid increase in apparent temperature. Before seven of the strokes (numbered 2, 3, 5, 8, 10, 13, and 14), the mean apparent temperatures in the control periods were significantly higher than during the hazard periods reflecting the strokes occurred after a rapid decrease in apparent temperature. Before five of the strokes (numbered 1, 6, 9, 12, and 15), there was no significant difference between the mean apparent temperature in the control period and the hazard period. Rapid, large changes in mean apparent temperature in either direction occurred in 10 out of the 15 observed canine strokes (67%), with an average change of 4 °C.

## 4. Discussion

The current findings demonstrate that the incidence of the canine ischemic strokes examined in the present study display an obvious peak in the fall season (September–November). In addition, the canine ischemic strokes evaluated tended to occur after periods of rapid fluctuations in weather conditions, including periods of both rising and declining temperature, atmospheric pressure, humidity, and apparent temperature. 

The canine ischemic strokes evaluated in the present study did display a seasonal peak, with over half of the stroke events occurring in the fall (September–November; Figure 1). The second quarter of the year (April, May, and June) has historically been the busiest for veterinarians and consequently, more MRIs are performed during this timeframe [16]. The slowest time of the year tends to be the last quarter (October, November, and December), where fewer MRIs are performed [16]. In the current study, fall was defined as September, October, and November—the time of the year where fewer MRIs are performed—suggesting that the incidence of ischemic stroke is indeed, likely to be greater during the fall. Previous studies have shown that pain intensity from osteoarthritis and sickle cell disease in humans, as well as circulating levels of C reactive protein (CRP; an acute phase inflammatory marker), exhibit peaks in the fall [40,41,42]. In addition, it has been demonstrated that the incidence of subarachnoid hemorrhage and acute myocardial infarction in humans also peaks in the fall [14,43]. Moreover, large variations in decreasing temperatures have been associated with an increased risk of ischemic stroke, especially in the fall [16]. Therefore, it would appear that seasonal occurrence of stroke in the canine is similar to the seasonal occurrence of some human diseases, including ischemic stroke.

Additionally, the current results demonstrate that the canine ischemic strokes evaluated tended to occur after periods of rapid fluctuations in weather conditions, including periods of both rising and declining temperature (Figure 2), atmospheric pressure (Figure 3), and humidity (Figure 4). It is commonly reported that it is the rapid change in weather conditions preceding stroke onset that may be the trigger [16,17,28,44,45,46]. In fact, it has been shown that sudden changes in temperature of up to 3–5 °C prior to stroke onset may be a potential trigger, and the results of the current study showed an average change in temperature of approximately 4.5 °C preceding the onset of the canine ischemic strokes evaluated [15,47]. In addition, the present study demonstrated average changes in humidity of approximately 15%, and average changes in atmospheric pressure of approximately 6.8 mmHg preceding the onset of the canine ischemic strokes evaluated. It has been shown that rapid changes in humidity of greater than 5%, and rapid changes in atmospheric pressure of up to 7.5 mmHg prior to stoke onset may be a potential trigger [16,17,28].

The current study also evaluated the apparent temperature calculation, which combines temperature, wind speed, and relative humidity into one measure. Combining several weather parameters in this fashion is thought to represent the overall environmental conditions on a particular day [15,17]. The current study demonstrated that the canine ischemic strokes evaluated tended to occur after periods of change in apparent temperature, regardless of direction (Figure 5), similar to that reported in other human studies utilizing the apparent temperature calculation [15,17,38].

The weather data used in the current study were collected from the National Centers for Environmental Information National Oceanic and Atmospheric Administration, which compiles data for this region from a single weather station at Boston Logan International Airport. This data obviously does not include individual environmental exposure or the effect of artificial heat and air conditioning, which is a significant confounder in many seasonality studies, including the current one [15,16,27,28,48,49,50,51,52]. It is difficult to include sufficient controls when studying the environmental impact of a naturally occurring disease, as this would be similar to the controlled laboratory studies that do not necessarily reflect spontaneous disease.

As stated previously, the small sample size utilized in the current study is an important limitation. Very large sample sizes are ideal to document effect modifiers, such as the effects of weather parameters on ischemic stroke risk and mortality, as has been demonstrated by studies utilizing anywhere from 200 to over 2,000,000 patients [15,16,28,32,53,54]. Although large sample sizes are considered ideal, there are a number of studies that have contributed to our knowledge of ischemic stroke triggers that have utilized fewer than 15 subjects per group [55,56,57,58,59,60], suggesting that the current study is an important addition to our knowledge base. In fact, some scientists have recently argued against the assumption that studies with small sample sizes are not scientifically justified [29,61,62].

## 5. Conclusions

Overall, the current study demonstrates that the incidence of the canine ischemic strokes examined display an obvious peak in the fall season (September–November), and the onset of those strokes tended to occur after periods of fluctuations in weather conditions, including periods of both rising and declining temperature, atmospheric pressure, humidity, and apparent temperature. These results are potentially clinically significant for a number of reasons. For example, if veterinarians are aware of an increased incidence of stroke during specific weather conditions, they may be more attentive to early clinical signs of canine stroke under certain weather conditions. Similarly, clients who own a dog at an increased risk of ischemic stroke or a dog that has had a previous stroke, may be more attentive to early signs of ischemic stroke if they are aware of certain weather conditions as a potential stroke trigger. Although the results of the present study should be interpreted with caution, they afford significant possibility for further investigation. Additional investigation into ischemic stroke triggers has the potential to further our fairly limited understanding of the overall pathogenesis of ischemic stroke. Furthermore, as there is currently no epidemiological data on the seasonal variability of ischemic stroke in dogs, the present study and any future studies documenting the relationship between various weather parameters and canine ischemic stroke, would further validate the use of ischemic stroke in the dog as an appropriate naturally occurring model.

## Figures and Tables

**Table 1:** Canine patient demographics.

Demographics	Number of Dogs	Percentage	Mean (Range)
Age (years)			10 (4–16)
Sex			
Male (neutered)	10 (9)	67%	
Female (spayed)	5 (5)	33%	
Weight (kg)			25 (5–52)
Breed			
Mixed Breed	3		
Labrador Retriever	2		
Rhodesian Ridgeback	2		
Boston Terrier	2		
Greyhound	2		
Australian Cattle Dog	1		
Australian Shepherd	1		
Bichon Frise	1		
Silky Terrier	1		

**Table 2:** Weather conditions at Boston Logan International Airport 8 days prior to the occurrence of 15 canine strokes.

Stroke Number	Date	Temperature (°C)		Atmospheric Pressure (mmHg)		Humidity (%)	
		M	SD	M	SD	M	SD
1	10 October 2011	14.58	4.92	767.14	4.82	62.50	15.98
2	25 July 2012	22.08	3.01	760.60	4.10	67.00	12.04
3	13 December 2013	−3.82	4.78	765.52	4.91	75.63	10.98
4	7 May 2014	10.21	2.70	759.56	6.32	64.13	18.15
5	19 May 2014	15.14	3.40	765.21	3.20	63.00	18.91
6	9 June 2014	18.47	2.52	759.78	3.18	67.88	15.12
7	13 June 2014	18.06	2.61	761.40	2.84	71.75	14.59
8	8 September 2014	21.81	21.81	763.40	3.95	71.00	8.02
9	16 September 2014	14.38	14.38	765.46	3.01	76.63	8.73
10	28 September 2014	16.11	16.11	766.44	7.03	72.13	8.08
11	29 September 2014	16.25	16.25	767.14	4.82	70.13	5.41
12	20 October 2014	13.26	13.26	760.60	4.10	78.50	9.80
13	22 October 2014	12.29	12.29	765.52	4.91	80.88	11.81
14	24 November 2014	1.67	1.67	757.49	8.89	59.50	14.96
15	6 January 2015	−4.65	−4.65	759.56	6.32	61.38	16.73

## References

[1] Donnan G.A., Fisher M., Macleod M., Davis S.M. (2008). Stroke. Lancet.

[2] Moskowitz M.A., Lo E.H., Iadecola C. (2010). The science of stroke: Mechanisms in search of treatments. Neuron.

[3] Broussalis E., Killer M., McCoy M., Harrer A., Trinka E., Kraus J. (2012). Current therapies in ischemic stroke. Part A. Recent developments in acute stroke treatment and in stroke prevention. Drug Discov. Today.

[4] Neuhaus A.A., Rabie T., Sutherland B.A., Papadakis M., Hadley G., Cai R., Buchan A.M. (2014). Importance of preclinical research in the development of neuroprotective strategies for ischemic stroke. JAMA Neurol..

[5] Fluri F., Schuhmann M.K., Kleinschnitz C. (2015). Animal models of ischemic stroke and their application in clinical research. Drug Des. Dev. Ther..

[6] Mozaffarian D., Benjamin E.J., Go A.S., Arnett D.K., Blaha M.J., Cushman M., Das S.R., De Ferranti S., Despres J.P., Fullerton H.J. (2016). Heart Disease and Stroke Statistics-2016 Update: A Report from the American Heart Association. Circulation.

[7] Durukan A., Tatlisumak T. (2007). Acute ischemic stroke: Overview of major experimental rodent models, pathophysiology, and therapy of focal cerebral ischemia. Pharmacol. Biochem. Behav..

[8] Altay U.M., Skerritt G.C., Hilbe M., Ehrensperger F., Steffen F. (2011). Feline cerebrovascular disease: Clinical and histopathologic findings in 16 cats. J. Am. Anim. Hosp. Assoc..

[9] Garosi L.S. (2010). Cerebrovascular Disease in Dogs and Cats. Vet. Clin. N. Am. Small Anim. Pract..

[10] Veterinary Information Network (VIN)—For Veterinarians, by Veterinarians. http://www.vin.com/.

[11] DeLahunta A., Glass E., Kent M. (2015). Veterinary Neuroanatomy and Clinical Neurology.

[12] Henderson V.W., Lobo R.A. (2012). Hormone therapy and the risk of stroke: Perspectives 10 years after the Women’s Health Initiative trials. Climact. J. Int. Menopause Soc..

[13] Kalogeris T., Baines C.P., Krenz M., Korthuis R.J. (2012). Cell biology of ischemia/reperfusion injury. Int. Rev. Cell Mol. Biol..

[14] Kelly-Hayes M., Wolf P.A., Kase C.S., Brand F.N., McGuirk J.M., D’Agostino R.B. (1995). Temporal patterns of stroke onset the Framingham Study. Stroke.

[15] Mostofsky E., Wilker E.H., Schwartz J., Zanobetti A., Gold D.R., Wellenius G.A., Mittleman M.A. (2014). Short-term changes in ambient temperature and risk of ischemic stroke. Cerebrovasc. Dis. Extra.

[16] Lichtman J.H., Leifheit-Limson E.C., Jones S.B., Wang Y., Goldstein L.B. (2016). Average Temperature, Diurnal Temperature Variation, and Stroke Hospitalizations. J. Stroke Cerebrovasc. Dis..

[17] Lian H., Ruan Y., Liang R., Liu X., Fan Z. (2015). Short-Term Effect of Ambient Temperature and the Risk of Stroke: A Systematic Review and Meta-Analysis. Int. J. Environ. Res. Public Health.

[18] Cowperthwaite M.C., Burnett M.G. (2011). An analysis of admissions from 155 United States hospitals to determine the influence of weather on stroke incidence. J. Clin. Neurosci..

[19] Awano T., Johnson G.S., Wade C.M., Katz M.L., Johnson G.C., Taylor J.F., Perloski M., Biagi T., Baranowska I., Long S. (2009). Genome-wide association analysis reveals a SOD1 mutation in canine degenerative myelopathy that resembles amyotrophic lateral sclerosis. Proc. Natl. Acad. Sci. USA.

[20] Coates J.R., Wininger F.A. (2010). Canine Degenerative Myelopathy. Vet. Clin. North Am. Small Anim. Pract..

[21] Crisp M.J., Beckett J., Coates J.R., Miller T.M. (2013). Canine degenerative myelopathy: Biochemical characterization of superoxide dismutase 1 in the first naturally occurring non-human amyotrophic lateral sclerosis model. Exp. Neurol..

[22] Gredal H., Skerritt G.C., Gideon P., Arlien-Soeborg P., Berendt M. (2013). Spontaneous ischaemic stroke in dogs: Clinical topographic similarities to humans. Acta Neurol. Scand..

[23] Hoffman J.M., Creevy K.E., Promislow D.E.L. (2013). Reproductive capability is associated with lifespan and cause of death in companion dogs. PLoS ONE.

[24] Kandel E.R. (2013). Principles of Neural Science.

[25] Kumar M.S.A. (2013). Clinically Oriented Anatomy of the Dog & Cat.

[26] National Oceanic and Atmospheric Administration|U.S. Department of Commerce. http://www.noaa.gov/.

[27] Hong Y.-C., Rha J.-H., Lee J.-T., Ha E.-H., Kwon H.-J., Kim H. (2003). Ischemic stroke associated with decrease in temperature. Epidemiology.

[28] Rakers F., Schiffnerm R., Rupprecht S., Brandstadt A., Witte O.W., Walther M., Schlattmann P., Schwab M. (2015). Rapid weather changes are associated with increased ischemic stroke risk: A case-crossover study. Eur. J. Epidemiol..

[29] Bacchetti P., Wolf L.E., Segal M.R., McCulloch C.E. (2005). Ethics and sample size. Am. J. Epidemiol..

[30] Halpern S.D., Karlawish J.H.T., Berlin J.A. (2002). The continuing unethical conduct of underpowered clinical trials. JAMA.

[31] Panter A.T., Sterba S.K. (2011). Handbook of Ethics in Quantitative Methodology.

[32] Gomes J., Damasceno A., Carrilho C., Lobo V., Lopes H., Madede T., Pravinrai P., Silva-Matos C., Diogo D., Azevedo A. (2015). Triggering of stroke by ambient temperature variation: A case-crossover study in Maputo, Mozambique. Clin. Neurol. Neurosurg..

[33] Gunes H., Kandis H., Saritas A., Dikici S., Buyukkaya R. (2015). The relationship between ischemic stroke and weather conditions in Duzce, Turkey. World J. Emerg. Med..

[34] Brandstätter E. (1999). Confidence intervals as an alternative to significance testing. Methods Psychol. Res. Online.

[35] Fidler F., Thomason N., Cumming G., Finch S., Leeman J. (2004). Editors can lead researchers to confidence intervals, but can’t make them think: Statistical reform lessons from medicine. Psychol. Sci..

[36] Nuzzo R. (2014). Scientific method: Statistical errors. Nature.

[37] Halsey L.G., Curran-Everett D., Vowler S.L., Drummond G.B. (2015). The fickle P value generates irreproducible results. Nat. Methods.

[38] Krstić G. (2011). Apparent temperature and air pollution vs. elderly population mortality in Metro Vancouver. PLoS ONE.

[39] Kalkstein L.S., Valimont K.M. (1986). An Evaluation of Summer Discomfort in the United State Using a Relative Climatological Index. Bull. Am. Meteorol. Soc..

[40] Chiriboga D.E., Ma Y., Li W., Stanek E.J., Hebert J.R., Merriam P.A., Rawson E.S., Ockene I.S. (2009). Seasonal and Sex Variation of High-Sensitivity C-Reactive Protein in Healthy Adults: A Longitudinal Study. Clin. Chem..

[41] McAlindon T., Formica M., Schmid C.H., Fletcher J. (2007). Changes in barometric pressure and ambient temperature influence osteoarthritis pain. Am. J. Med..

[42] Smith W.R., Bauserman R.L., Ballas S.K., McCarthy W.F., Steinberg M.H., Swerdlow P.S., Waclawiw M.A., Barton B.A., The Investigators of the Multicenter Study of Hydroxyurea in Sickle Cell Anemia (2009). Climatic and geographic temporal patterns of pain in the Multicenter Study of Hydroxyurea. Pain.

[43] Houck P.D., Lethen J.E., Riggs M.W., Gantt D.S., Dehmer G.J. (2005). Relation of atmospheric pressure changes and the occurrences of acute myocardial infarction and stroke. Am. J. Cardiol..

[44] Kyobutungi C., Grau A., Stieglbauer G., Becher H. (2005). Absolute temperature, temperature changes and stroke risk: A case-crossover study. Eur. J. Epidemiol..

[45] Coelho F.M.S., Santos B.F.C.D., Neto M.C., Lisboa L.F., Cypriano A.S., Lopes T.O., de Miranda M.J., Avila M.H., Alonso J.B., Pinto H.S. (2010). Temperature variation in the 24 hours before the initial symptoms of stroke. Arq. Neuropsiquiatr..

[46] Zhang X.-J., Ma W.-P., Zhao N.-Q., Wang X.-L. (2016). Time series analysis of the association between ambient temperature and cerebrovascular morbidity in the elderly in Shanghai, China. Sci. Rep..

[47] Gomes J., Damasceno A., Carriho C., Lobo V., Lopes H., Madede T., Pravinrai P., Silca-Matos C., Diogo D., Azevedo A. (2014). The effect of season and temperature variation on hospital admissions for incident stroke events in Maputo, Mozambique. J. Stroke Cerebrovasc. Dis..

[48] Magalhães R., Silva M.C., Correia M., Bailey T. (2011). Are stroke occurrence and outcome related to weather parameters? Results from a population-based study in northern portugal. Cerebrovasc. Dis..

[49] Çevik Y., Doğan N.Ö., Daş M., Ahmedali A., Kul S., Bayram H. (2015). The association between weather conditions and stroke admissions in Turkey. Int. J. Biometeorol..

[50] Goggins W.B., Woo J., Ho S., Chan E.Y.Y., Chau P.H. (2012). Weather, season, and daily stroke admissions in Hong Kong. Int. J. Biometeorol..

[51] Halonen J.I., Zanobetti A., Sparrow D., Vokonas P.S., Schwartz J. (2011). Outdoor temperature is associated with serum HDL and LDL. Environ. Res..

[52] Zheng D., Arima H., Heeley E., Karpin A., Yang J., Chalmers J., Anderson C.S., The INTERACT investigators (2015). Ambient temperature and volume of perihematomal edema in acute intracerebral haemorrhage: The INTERACT1 study. Int. J. Stroke.

[53] Lichtman J.H., Jones S.B., Wang Y., Leifheit-Limson E.C., Goldstein L.B. (2013). Seasonal variation in 30-day mortality after stroke: Teaching versus nonteaching hospitals. Stroke J. Cereb. Circ..

[54] Roberson S., Dutton M., Macdonald M., Odoi A. (2016). Does Place of Residence or Time of Year Affect the Risk of Stroke Hospitalization and Death? A Descriptive Spatial and Temporal Epidemiologic Study. PLoS ONE.

[55] Kanikowska D., Sato M., Iwase S., Shimizu Y., Nishimura N., Inukai Y., Sugenoya J. (2013). Effects of living at two ambient temperatures on 24-h blood pressure and neuroendocrine function among obese and non-obese humans: A pilot study. Int. J. Biometeorol..

[56] Kanikowska D., Sugenoya J., Satom M., Shimizu Y., Inukai Y., Nishimura N., Iwase S. (2010). Influence of season on plasma antidiuretic hormone, angiotensin II, aldosterone and plasma renin activity in young volunteers. Int. J. Biometeorol..

[57] Kirsz K., Szczesna M., Molik E., Misztal T., Wojtowicz A.K., Zieba D.A. (2012). Seasonal changes in the interactions among leptin, ghrelin, and orexin in sheep. J. Anim. Sci..

[58] Kruse H.-J., Wieczorek I., Hecker H., Creutzig A., Schellong S.M. (2002). Seasonal variation of endothelin-1, angiotensin II, and plasma catecholamines and their relation to outside temperature. J. Lab. Clin. Med..

[59] Sato M., Kanikowska D., Sugenoya J., Inukai Y., Shimizu Y., Nishmura N., Iwase S. (2011). Effects of aging on thermoregulatory responses and hormonal changes in humans during the four seasons in Japan. Int. J. Biometeorol..

[60] Zhu W.L., Wang Z.K. (2015). Seasonal changes in body mass, serum leptin levels and hypothalamic neuropeptide gene expression in male *Eothenomys olitor*. Comp. Biochem. Physiol. A Mol. Integr. Physiol..

[61] Bacchetti P. (2013). Small sample size is not the real problem. Nat. Rev. Neurosci..

[62] Quinlan P.T. (2013). Misuse of power: In defence of small-scale science. Nat. Rev. Neurosci..

